# Role of Cyclic Nucleotide-Dependent Actin Cytoskeletal Dynamics: [Ca^2+^]_i_ and Force Suppression in Forskolin-Pretreated Porcine Coronary Arteries

**DOI:** 10.1371/journal.pone.0060986

**Published:** 2013-04-12

**Authors:** Kyle M. Hocking, Franz J. Baudenbacher, Gowthami Putumbaka, Sneha Venkatraman, Joyce Cheung-Flynn, Colleen M. Brophy, Padmini Komalavilas

**Affiliations:** 1 Department of Surgery, Vanderbilt University Medical Center, Nashville, Tennessee, United States of America; 2 Department of Biomedical Engineering, Vanderbilt University, Nashville, Tennessee, United States of America; 3 VA Tennessee Valley Healthcare System, Nashville, Tennessee, United States of America; University of Illinois, Urbana-Champaign, United States of America

## Abstract

Initiation of force generation during vascular smooth muscle contraction involves a rise in intracellular calcium ([Ca^2+^]_i_) and phosphorylation of myosin light chains (MLC). However, reversal of these two processes alone does not account for the force inhibition that occurs during relaxation or inhibition of contraction, implicating that other mechanisms, such as actin cytoskeletal rearrangement, play a role in the suppression of force. In this study, we hypothesize that forskolin-induced force suppression is dependent upon changes in actin cytoskeletal dynamics. To focus on the actin cytoskeletal changes, a physiological model was developed in which forskolin treatment of intact porcine coronary arteries (PCA) prior to treatment with a contractile agonist resulted in complete suppression of force. Pretreatment of PCA with forskolin suppressed histamine-induced force generation but did not abolish [Ca^2+^]_i_ rise or MLC phosphorylation. Additionally, forskolin pretreatment reduced filamentous actin in histamine-treated tissues, and prevented histamine-induced changes in the phosphorylation of the actin-regulatory proteins HSP20, VASP, cofilin, and paxillin. Taken together, these results suggest that forskolin-induced complete force suppression is dependent upon the actin cytoskeletal regulation initiated by the phosphorylation changes of the actin regulatory proteins and not on the MLC dephosphorylation. This model of complete force suppression can be employed to further elucidate the mechanisms responsible for smooth muscle tone, and may offer cues to pathological situations, such as hypertension and vasospasm.

## Introduction

Smooth muscle activation of the H1 receptor via histamine is linked to the intracellular G protein (Gαq) and Gαq-coupled receptors that activate phospholipase C (PLC) and RhoA (reviewed in [1]). Activation of PLC induces inositol 1, 4, 5-trisphosphate (IP_3_) production, causing calcium to be released from the sarcoplasmic reticulum (SR). This increase in intracellular calcium activates calmodulin-dependent myosin light chain kinase (MLCK), leading to increases in the phosphorylation of 20 KDa myosin light chains (MLC). Crossbridge phosphorylation of the actomyosin apparatus results in the generation of force in vascular smooth muscle [2], [3], [4]. While Ca^2+^ and MLC phosphorylation are important for the initiation of contraction, the tonic phase, or force maintenance, of smooth muscle contraction can occur where [Ca^2+^]_i_ levels and MLC phosphorylation are near basal levels, suggesting other pathways are engaged during force maintenance in smooth muscle [2], [5], [6], [7], [8], [9]. During the sustained phase of contraction, stiffness and force are maintained at high levels while Ca^2+^_,_ crossbridge phosphorylation, and shortening velocity fall to intermediate values [6], [10], [11], [12]. Maintenance of high force despite intermediate levels of crossbridge phosphorylation and velocity was explained to be due to the latch phenomenon [13], [14]. Other investigators have suggested that force maintenance is due to the regulation of ADP association with muscle fibers [15]. More recently, actin cytoskeletal dynamics have been implicated in the modulation of vascular smooth muscle tone [16], [17]. Similarly, sustained phase of swine carotid artery contraction was associated with increased paxillin (Y118) phosphorylation and actin polymerization [18].

Vascular smooth muscle relaxation, or inhibition of force, can be mediated by vasodilators that activate guanylyl cyclase (e.g. nitric oxide) or adenylyl cyclase (e.g. prostacyclin, β-agonists, and forskolin), leading to increases in cGMP and cAMP, respectively. The cyclic nucleotides, in turn, activate cGMP-dependent protein kinase (PKG) and cAMP-dependent protein kinase (PKA) [19], leading to several phosphorylation events resulting in relaxation or inhibition of force. Cyclic nucleotide-induced relaxation or inhibition of force in smooth muscle involves at least three major pathways: decreases in intracellular free calcium concentrations, calcium sensitivity and actin cytoskeletal regulation (reviewed in [19], [20]). While the role of a decrease in [Ca^2+^]_i_ and Ca^2+^ sensitivity in the regulation of smooth muscle cell contraction has been established, the role of actin cytoskeleton and actin-associated proteins is still unclear. Although several investigations have suggested the regulation of actin and actin-associated proteins in smooth muscle contraction (reviewed in [17]), very few reports have addressed the role of second messenger regulation of actin-associated proteins during inhibition of force. Actin-associated proteins that are implicated in the regulation of smooth muscle contraction include the small heat shock-related protein 20 (HSP20 or HSPB6), cofilin, vasodilator-stimulated phosphoprotein (VASP) and paxillin. HSP20 is an actin binding protein that is phosphorylated by PKG and PKA on serine 16, inducing relaxation and inhibition of contraction through the modulation of actin cytoskeletal dynamics [21], [22], [23], [24]. Cyclic nucleotide-dependent relaxation is associated with decreases in the phosphorylation of the actin depolymerizing protein cofilin in vascular, as well as, airway smooth muscle cells [24], [25]. VASP is an actin binding protein that is localized to focal adhesions and cell-to-cell contacts [26]. Forskolin treatment leads to phosphorylation of VASP and regulates the actin cytoskeleton in rat aortic smooth muscle cells [27] and human airway smooth muscle cells [25]. Paxillin is a scaffolding protein that serves as a multi-domain adaptor at the interface between the plasma membrane and the actin cytoskeleton (reviewed in [28]). Paxillin undergoes phosphorylation and activation in response to contractile stimulation in many smooth muscle tissue types and is involved in the regulation of actin polymerization during contraction [18], [29].

In this study, we hypothesized that cyclic nucleotide-induced force suppression is dependent upon changes in actin cytoskeletal dynamics. We developed a physiological model in which histamine-induced force was completely suppressed by forskolin pretreatment in PCA smooth muscle. In this novel approach, force and [Ca^2+^]_i_ were measured concurrently using a FluoroPlex Tissue Bath Fluorometry System, and polymerization of actin, phosphorylation of actomyosin and actin-associated proteins were determined. Our results indicate that forskolin-induced suppression of force does not completely abolish Ca^2+^ transients or MLC phosphorylation but causes depolymerization of actin dose-dependently, and changes in the phosphorylation of proteins such as HSP20, cofilin, VASP and paxillin that regulate actin polymerization. Since force can be completely suppressed without abolishing [Ca^2+^]_i_, this model system can be employed to elucidate as yet unidentified Ca^2+^-independent molecular determinants of force inhibition in vascular smooth muscle.

## Materials and Methods

### Materials

All chemicals were purchased from Sigma Chemical Co. (St. Louis, MO) unless specified otherwise. Pre-cast acrylamide gels, Sodium dodecyl sulfate (SDS), Tris-glycine-SDS buffer (TGS), Tris-glycine (TG) and prestained Precision Blue Protein Standards were purchased from Bio-Rad (Hercules, CA). Urea and CHAPS (3-[(3-Cholamidopropyl) dimethylammonio]-1-propanesulfonate) were from Research Organics Inc. (Cleveland, OH). F/G Actin assay kit was from Cytoskeleton Inc., (Denver, CO). Fura 2-AM and Pluronic F-127 was purchased from Invitrogen (Carlsbad, CA).

### Procurement of Porcine Coronary Artery Smooth Muscle Tissue and Physiologic Measurements

Fresh cadaveric hearts were isolated immediately from euthanized, discarded animals from Vanderbilt University Medical Center. All procedures for collection of cadaveric tissue were reviewed and approved by the Vanderbilt University Animal Care and Use Committee. Porcine hearts were also collected from the local slaughter house (C&F Meats, Triune, TN) after obtaining permission from the slaughter house to use the tissue for research. The heart was procured and placed directly in HEPES buffer (140 mM NaCl, 4.7 mM KCl, 1.0 mM MgSO_4_, 1.0 mM NaH_2_PO_4_, 1.5 mM CaCl_2_, 10 mM glucose, and 10 mM HEPES, pH 7.4), and the coronary arteries were dissected and tested immediately or after overnight storage at 4°C in University of Wisconsin solution. Subcutaneous fat and adventitial tissues were removed and the vessel was cut into transverse rings of 3.0 mm in width. To focus on the smooth muscle-derived changes during inhibition of force, endothelium was denuded by gently rolling the luminal surface of each ring at the tip of a fine forceps. Rings were suspended in a muscle bath containing a bicarbonate buffer (120 mM NaCl, 4.7 mM KCl, 1.0 mM MgSO_4_, 1.0 mM NaH_2_PO_4_, 10 mM glucose, 1.5 mM CaCl_2_, and 25 mM Na_2_HCO_3_, pH 7.4), equilibrated with 95% O_2_/5% CO_2_, at 37°C. Force measurements were obtained with either a Kent Scientific (Litchfield, CT) force transducer (TRN001) or a Radnoti force transducer (Radnoti Glass Technology Inc., Monrovia, CA) interfaced with Power Lab from AD Instruments (Colorado Springs, CO). Data were recorded with Chart software, version 5.1.1 (AD Instruments). Rings were washed every 15 min with 37°C bicarbonate buffer for 1 hr, and each ring was progressively stretched to its optimal resting tension (approximately 1 g) that would produce a maximal response to contractile agonists as determined previously, then maintained at the resting tension and equilibrated for another hour. Rings were then contracted multiple times with high extracellular potassium (110 mM KCl, with equimolar replacement of NaCl in bicarbonate buffer) and the force generated was measured. Measured force was normalized for ring weight and length and converted to Stress using the formula: Stress [10^5^Newtons (N)/m^2^] = force (g) × 0.0987/area, where area is equal to the wet weight [mg/length (mm at maximal length)] divided by 1.055. The maximal tension obtained was taken as 100%. Rings were equilibrated for an additional 30 min and dose response curves for histamine contraction and forskolin relaxation were determined to select the correct dose of agents for the experiment. To determine the inhibition of contraction, rings were either treated with buffer alone (control), histamine (5 µM ) for 3 min, forskolin (5 µM) for 10 min, or forskolin (1, 5, or 10 µM) for 10 min followed by histamine (5 µM) for 3 min. At the end of the experiments, all rings were washed and contracted with KCl to ensure continued viability of the tissues. To determine the role of phosphorylation of proteins during inhibition of force, physiologic experiments were conducted as described above and the tissues were snap frozen under tension using forceps precooled in liquid nitrogen at 3 min and then pulverized. These pulverized tissues were stored at −80°C for later analysis using urea glycerol gel, SDS polyacrylamide gel electrophoresis (PAGE) or isoelectric focusing and western blotting. For actin assay to determine the level of F-actin compared to G-actin, the tissues were used immediately after treatment without freezing.

### Cytosolic [Ca^2+^]_i_ Measurements

Rings of PCA were suspended on hooks in a FluoroPlex Tissue Bath Fluorometry System (IonOptix LLC, Milton, MA and Radnoti Glass Technology Inc., Monrovia, CA), which enables fluorescence ion recording in parallel with force measurement. Force measurements were obtained with a Radnoti force transducer (Radnoti Glass Technology Inc., Monrovia, CA) interfaced with Power Lab from AD Instruments (Colorado Springs, CO). Rings were loaded at room temperature with 10 µM Fura-2 AM ester and 0.01% Pluronic F-127 in the bicarbonate buffer for 4 hrs. After loading, rings were washed every 10 min with 37°C bicarbonate buffer for 1 hr. [Ca^2+^]_i_ was measured with optical fibers that were interfaced with Power Lab. Fluorescence was measured at both 380 and 340 nm of wavelength, simultaneously. The ratio of the emission of the two wavelengths was used to determine intracellular changes in calcium concentration. Baseline ratio was set at 1.0 and changes in this ratio in response to stimuli were measured. Baseline calcium fluorescence was measured and the background was set to zero as an output of 1 volt. To determine the inhibition of contraction, rings were either treated with forskolin (1, 5, or 10 µM for 10 min), followed by histamine (5 µM) or histamine alone. To add forskolin or histamine while continuously measuring intracellular calcium concentrations, an infusion line filled with bicarbonate buffer was used to keep the system in a closed light impenetrable state. The amount of buffer in the infusion line was adjusted to achieve the final concentration of the agonist in the bath. Force and calcium fluorescence were measured continuously for 15 min after the addition of histamine.

### Determination of VASP, Cofilin and Paxillin Phosphorylation

Proteins from frozen muscle rings were extracted in UDC buffer (8 M urea, 10 mM dithiothreitol (DTT), 4% CHAPS containing protease inhibitor, Phosphatase I and II inhibitor cocktail (Sigma, St. Louis, MO). The mixtures were vortexed at room temperature overnight, and then centrifuged at 14,000 rpm for 15 min at 4°C. Soluble protein concentrations were determined using the Bradford assay (Pierce Chemical, Rockfort, IL). Equal amounts (20–50 µg) of proteins were placed in a Laemmli sample buffer (Bio-Rad laboratories, Inc. Hercules, CA), heated for 5 min at 100°C and separated on SDS polyacrylamide gels. Proteins from the gels were transferred onto nitrocellulose membranes (Li-COR Biosciences, Lincoln, NE) and blocked prior to incubation overnight at 4°C with the following primary antibodies: anti-VASP (1∶2000, ECM Biosciences, Versailles, KY); anti-phospho-cofilin 2 (Ser 3) (1∶500), and anti-cofilin (1∶500, Cell Signaling Technology, Santa Cruz, CA), anti phospho (Tyr 118)-paxillin (1∶250, Santa Cruz Biotechnology, Inc.) and anti-paxillin (1∶250, BD Transduction Laboratories). VASP phosphorylation by PKA at Ser 157 causes a significant mobility shift on one dimensional SDS-PAGE gels [30] enabling to separate phospho(p) and non-phospho(np) VASP on the same gel and was detected by using an antibody that recognizes both phospho and non-phospho forms. Membranes were washed three times with TBS containing Tween 20 (0.1%) (TBST), and incubated with appropriate infrared-labeled secondary antibodies (Li-Cor, Lincoln, NE) for 1 h at room temperature. The membranes were subsequently washed with TBST, and protein-antibody complexes were visualized and quantified using the Odyssey direct infrared fluorescence imaging system (Li-Cor Biosciences NE). Phosphorylation was calculated as a ratio of the phosphorylated protein to total protein (p- plus np- protein) and was then normalized to the unstimulated control with the control value set as 1.0.

### Determination of MLC Phosphorylation

MLC phosphorylation was determined using a modification of an established urea glycerol method that separates p- and np- MLC [31], [32]. The frozen tissue was pulverized, placed in a frozen slurry of precipitating solution consisting of 90% acetone, 10% trichloroacetic acid, and 10 mM DTT, and then allowed to melt to room temperature. The precipitating solution was removed, and the tissues were washed three times with 90% acetone and 10 mM DTT. The samples were dried, and the pellets were suspended in UDC buffer as described above and vortexed to solubilize the proteins. Ten micrograms of protein were diluted with 10 µl of urea sample buffer (6.7 M urea, 18 mM Tris, 20 mM glycine, 9 mM DTT, 4.6% saturated sucrose, and.004% bromophenol blue) and separated on glycerol-urea mini gels (40% glycerol, 10% acrylamide, 0.5% bisacrylamide, 20 mM Tris, and 22 mM glycine). Proteins were transferred onto nitrocellulose membranes in a buffer containing 10 mM Na_2_HPO_4_ pH 7.6 at 25 V for 1 hr at 20°C. The blot was probed with anti MLC antibody (1∶7000, gift from Dr. James Stull, University of Texas, Galveston TX), and processed as described above. The p- and np- MLC bands were quantitated by densitometric analysis. The relative amount of the p-MLC was calculated.

### Determination of HSP20 Phosphorylation

Phosphorylation of HSP20 in response to forskolin was examined by isoelectric focusing, which separates the p- and np- forms of HSP20 and detected by western blotting. 30 µg of extracted proteins from the treated PCA samples were separated on one-dimensional isoelectric focusing gel (8.3×7.3 cm) with 5% ampholines (4 parts pI 4–7 and 1 part pI 3–10, GE Healthcare Bio-Sciences) using 20 mM sodium hydroxide as a cathode buffer and 10 mM phosphoric acid as an anode buffer. Proteins were focused for 100 V for 1 hr, 250 V for 1 hr and 500 V for 30 min and transferred to nitrocellulose membrane at 25 V in 0.7% acetic acid with the direction of the gel sandwich reversed (acetic acid give proteins a positive charge) for 1 hr at room temperature. The blot was probed with anti-HSP20 antibody (1∶3,000 dilution, Advanced Immunochemical Inc., Long Beach, CA); and the p- and np- forms of HSP20 were quantitated by densitometry and the ratio of p-HSP20 to total HSP20 was calculated.

### Actin Assay

The amount of F-actin versus G-actin was measured using the G-actin/F-actin *In Vivo* Assay kit (Cytoskeleton, Denver, CO), per manufacturer’s protocol. Briefly, treated PCA samples were homogenized in 1 ml of lysis buffer (50 mM PIPES pH 6.9, 50 mM NaCl, 5 mM MgCl_2_ 5 mM EGTA, 5% (v/v) Glycerol, 0.1% Nonidet P40, 0.1% Triton X-100, 0.1% Tween 20, 0.1% 2-mercapto-ethanol, 0.001% Antifoam C, 4 µM Tosyl arginine methyl ester, 15 µM Leupeptin, 10 µM Pepstatin A, 10 mM Benzamidine, 1 mM ATP warmed to 37°C) for 1 min with a mortar and pestle that fit into the 1.5 ml microfuge tube. The lysate was centrifuged at 2000 rpm for 5 min at 37°C to pellet unbroken cells. The supernatants were centrifuged at 100,000×g for 1 hr at 37°C. Supernatants (contains the G-actin) were transferred to pre-cooled tubes and placed on ice. The pellets (contain F-actin) were resuspended in 1 ml of ice-cold 10 µM cytochalasin D in deionized water, and F- actin was depolymerized by incubating for 1 hr on ice with mixing every 15 min. Equal volume of supernatants and pellets along with actin standards (2–20 µg) were separated on 12% SDS-polyacrylamide gels and transferred to nitrocellulose membrane in 1X TG buffer at 100 volts for 1 hr. The membrane was probed with anti actin antibody and the amount of actin in each fraction was quantified comparing to actin standards loaded on the same gel.

### Statistical Analysis

All data are reported as the mean responses ± standard error of the mean (SEM). Statistical analysis was performed by unpaired Student’s *t* test or one-way *ANOVA,* followed by Tukey’s post test (GraphPad Software, Inc. San Diego, CA). The criterion for significance was p<0.05.

## Results

### The Effect of Forskolin on Inhibition of Contraction, Ca^2+^ Transients, and MLC Phosphorylation

**Figure 1:**
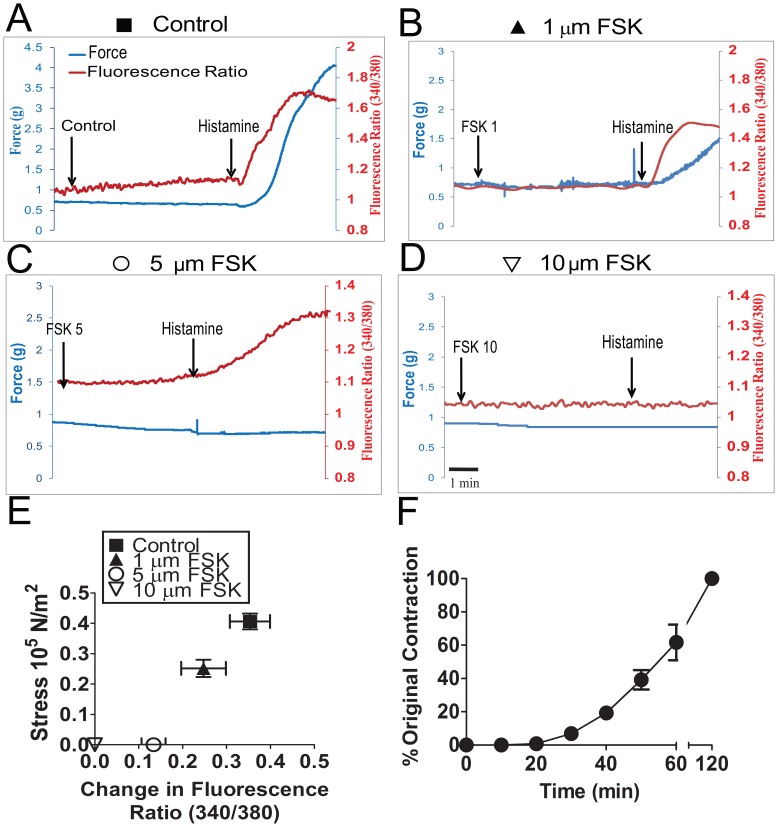
Preincubation with forskolin suppressed histamine-induced force in porcine coronary artery. PCA rings were suspended in a muscle bath and equilibrated in Kreb’s bicarbonate buffer for 2 h. Panel A–D: Representative force and intracellular calcium tracings of rings pretreated with either nothing, 1, 5, or 10 µM forskolin followed by 5 µM histamine. Panel E: Comparison of concurrent change in flourescent ratio (340/380 nm) to Stress (N/m^2^) from histamine-induced contraction with various doses of forskolin added as a pretreatment. Panel F: Reversibility of Forskolin-mediated force suppression. Contraction to 5 µM histamine was assesed after washing off the 5 µM forskolin. The sample was rechallanged with histamine every 10 min for the first 50 min to determine recovery of contraction. Samples were also challenged after two hours of washing which resulted in the full recovery of histamine induced contraction.

**Figure 2:**
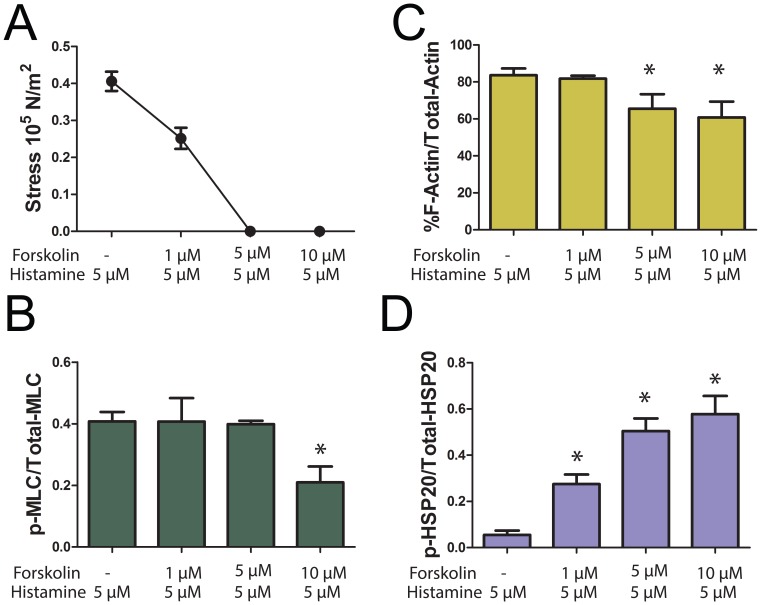
Dose dependent effect of forskolin on force, MLC phosphorylation, F-actin levels and HSP20 phosphorylation during suppression of force. PCA rings were suspended in a muscle bath and equilibrated in Kreb’s bicarbonate buffer for 2 h and rings were pretreated with either nothing, 1, 5, or 10 µM forskolin followed by 5 µM histamine. Force was measured and the tissue was frozen and analysed for MLC phosphorylation, F-actin levels, and HSP20 phosphorylation, Panel A: Cumulative data obtained when the force was converted to stress. Panel B: Cumulative data representing the relative amounts of the p-MLC over the total MLC obtained when the phosphorylated and non-phosphorylated bands were quantitated densitometrically. Panel C: F-actin concentration was calculated and cumulative data is plotted. Panel D: Cumulative data representing the relative amounts of p-HSP20 over total HSP20, obtained when the phosphorylated and non-phosphorylated bands were quantitated densitometrically.

To study the role of actin cytoskeletal dynamics during inhibition of contraction we first developed a physiological model system with conditions in which agonist-induced force was completely suppressed in the presence of Ca^2+^ transients with no significant change in the MLC phosphorylation. This allowed the study of putative mechanisms, other than calcium desensitization, that regulate the suppression of force. Initial experiments were conducted using various doses of histamine (0.1 to 10 µM) to contract PCA, and a dose of histamine (5 µM) which produced greater than 60% of maximal potassium-induced contraction was selected for our experiment (Figure 1 A). Treatment of PCA with histamine (5 µM) alone induced force (62% of KCl stress, Figure 1 A, E) and increased Ca^2+^ transients (0.35±0.05 AU, n = 9) (Figure 1 A, E). Changes in Ca^2+^ transients occurred before the initiation of contraction, and the fluorescence ratio reached a maximum value while force was still increasing. The maximum fluorescence ratio sustained for 45 seconds and dropped prior to any decrease in force. To develop a physiological model system with force completely suppressed in the presence of Ca^2+^ transients with no significant change in the MLC phosphorylation, we pretreated PCA with different doses of forskolin (1, 5, and 10 µM) for 10 min followed by histamine (5 µM) for 3 min and force, [Ca^2+^]_i_, MLC phosphorylation, F-actin levels and HSP20 phosphorylation were measured. Pretreatment of PCA with forskolin at 1 µM did not abolish histamine-induced force (38% of KCl stress, Figure 1B, E), [Ca^2+^]_i_ (0.25±0.05 AU, n = 7) (Figure 1 B, E), MLC phosphorylation (0.41±.03 and 0.41±0.08 p-MLC/total MLC for histamine and 1 µM forskolin plus histamine, respectively, Figure 2B) or significantly change the F-actin concentration (84±4%and 82±2% for histamine and forskolin plus histamine, respectively, Figure 2C). However, pretreatment with 1 µM forskolin increased the phosphorylation of HSP20 (0.06±0.02 and 0.28±0.04 p-HSP20/total HSP20 for histamine and forskolin plus histamine, respectively, Figure 2D). Forskolin at 5 µM completely suppressed histamine-induced force (0% of KCl stress, Figure 1B, E, Figure 2A) but did not abolish [Ca^2+^]_i_ (0.13±0.03 AU, n = 9) (Figure 1 C, E). The magnitude of the change in fluorescence ratio was significantly greater for tissue contracted with histamine (0.35±0.05 AU) when compared to tissue treated with 5 µM forskolin followed by histamine (0.13±0.03 AU)(n = 7, p<0.01, Figure 1E). The time it took for the [Ca^2+^]_i_ to rise from 10% to 90% of its maximal was significantly shorter (54.40±8.23 seconds) for tissue that was treated with histamine alone compared to forskolin followed by histamine treatment (192.8±45.63 seconds n = 5, p = 0.024, data not shown). Forskolin at 5 µM did not significantly change MLC phosphorylation (0.4.±0.03 and 0.39±.01 p-MLC/total MLC for histamine and forskolin plus histamine, respectively, Figure 2B), however it decreased F-actin levels (84±4% and 66±8% for histamine and forskolin plus histamine, respectively, Figure 2C) and increased the phosphorylation of HSP20 (0.06±0.02 and 0.50±.0.05 p-HSP20/total HSP20 for histamine and forskolin plus histamine, respectively, Figure 2D). Forskolin at 10 µM completely suppressed histamine-induced force (0% of KCl stress, Figure 1 D, E) as well as [Ca^2+^]_i_ (0 AU, n = 7) (Figure 1D, E). Forskolin at 10 µM significantly decreased the MLC phosphorylation (0.4.±0.03 and 0.21.±0.05 p-MLC/total MLC for histamine and forskolin plus histamine, respectively, Figure 2B), and F-actin levels (84±4% and 60±9% for histamine and forskolin plus histamine, respectively, Figure 2C) while it increased the phosphorylation of HSP20 (0.06±0.02 and 0.50±.0.05 p-HSP20/total HSP20 for histamine and forskolin plus histamine, respectively, Figure 2D). Hence, 5 µM forskolin was chosen for our model system and using these conditions we further performed experiments to characterize the effect of forskolin-induced suppression of force on phosphorylation changes of actin associated proteins and compared to basal levels. Forskolin-induced suppression of force was reversible as washing the rings repeatedly for 50 min allowed the PCA to contract (∼40% of original contraction) to 5 µM histamine in a time-dependent manner (Figure 1F), and demonstrated that the doses of histamine and forskolin used in this study did not affect the viability of the tissue. Washing the rings for 2 hr allowed the PCA to recover completely and produced 100% of the original contraction to 5 µM histamine.

**Figure 3:**
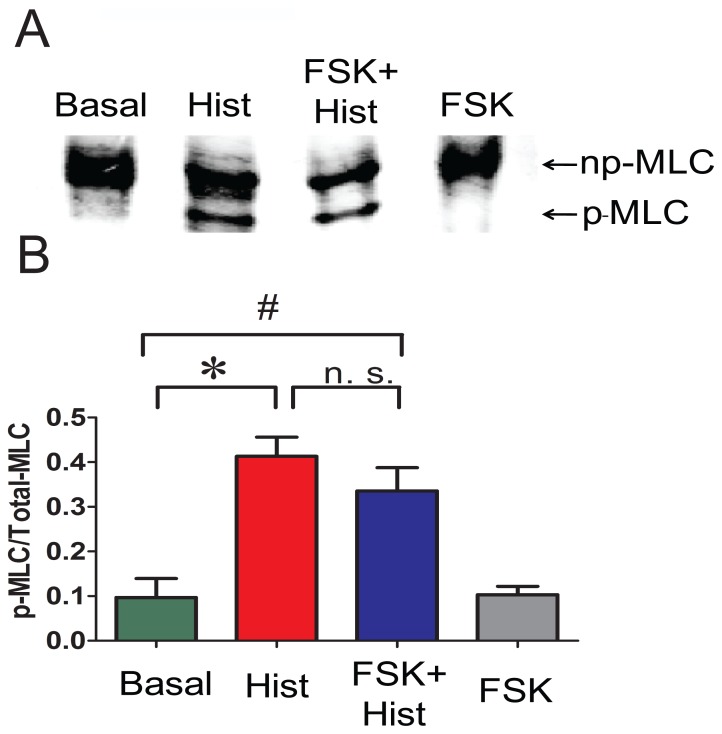
Preincubation with forskolin does not abolish histamine-induced increases in MLC phosphorylation. PCA rings were suspended in a muscle bath and equilibrated in Kreb’s bicarbonate buffer for 2 h. Rings were treated with either basal conditions, 5 µM histamine for 3 min, 5 µM forskolin for 10 min followed by 5 µM histamine for 3 min, or 5 µM forskolin for 10 min and snap frozen. Proteins were extracted and loaded on urea glycerol gels to separate the p-MLC and np-MLC and were detected by western blotting using an antibody that recognizes both forms. Panel A: Representative western blot of p-MLC and np-MLC. Panel B: Cumulative data representing the relative amounts of the p-MLC over the total MLC obtained when the phosphorylated and non-phosphorylated bands were quantitated densitometrically n = 4. *significance between Histamine and Basal. ^#^significance between Forskolin plusHistamine and Basal.

Histamine treatment significantly increased the phosphorylation of MLC (0.41±0.04 p-MLC/total MLC) when compared to untreated basal (0.09±0.04 p-MLC/total MLC) (n = 4, p = 0.01). Forskolin plus histamine treatment also significantly increased (0.34±0.05 p-MLC/total MLC the phosphorylation of MLC when compared to untreated basal (0.09±0.04 p-MLC/total MLC, n = 4, p = 0.02). There was no significant difference in the MLC phosphorylation between histamine and forskolin plus histamine treated tissues (Figure 3), which suggests that forskolin treatment did not result in significant dephosphorylation of MLC.

### Forskolin Treatment Decreases Filamentous Actin Levels

**Figure 4:**
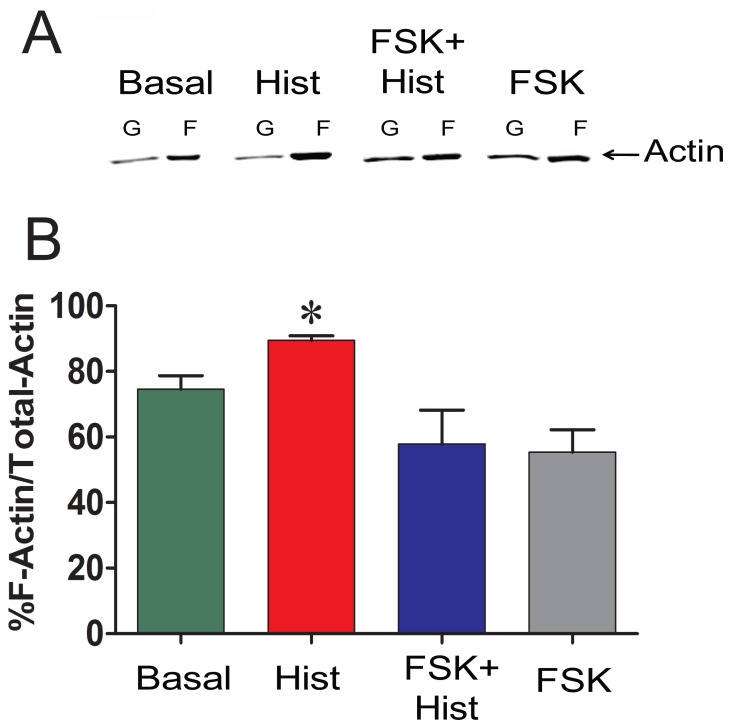
Preincubation with forskolin prevent histamine-induced increases in F-actin. PCA rings were treated the same way as described in Figure 3. Panel A: Treated tissues were homogonized and F- and G-actin were separated by centrifugation, F-actin was converted to G-actin, and measured by western blot, and quantitated compared to the standard actin loaded on the same gel. Panel B: F-actin concentration was calculated and cumulative data is plotted n = 7, p<0.05. *significance between Histamine and all other groups.

Several investigators have demonstrated that actin is polymerized during contraction of smooth muscle, and agents that inhibit actin polymerization result in inhibition of contraction (reviewed in [17]). To examine the effect of activation of cyclic nucleotide-dependent pathways by forskolin on actin polymerization, actin polymerization was measured in PCA contracted with histamine with or without forskolin pretreatment (Figure 4). Treatment with histamine led to increases in F- actin by 14% (75±4% to 89±1% for basal and histamine, respectively), while treatment with forskolin reduced F-actin by 10% (75±4% to 65±6% for basal and forskolin, respectively). Pretreatment with forskolin before histamine stimulation reduced the F-actin by 17% (75±4% to 58±10% for basal and forskolin plus histamine, respectively p = 0.004, n = 7–9).

### The Effect of Forskolin on the Phosphorylation of Actin Regulating Proteins, HSP20, Cofilin, VASP, and Paxillin

**Figure 5:**
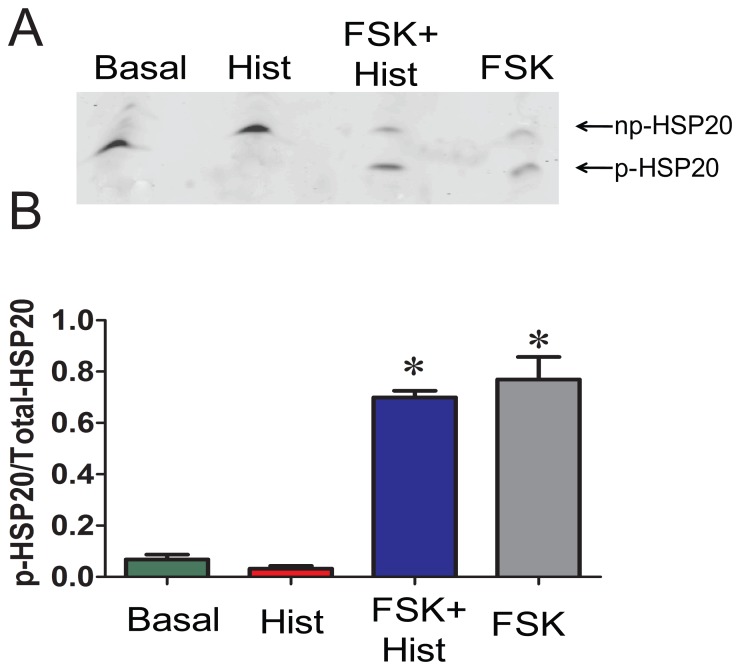
Preincubation with forskolin induces phosphorylation of HSP20. Treatment conditions are the same as described in Figure 3. Proteins were extracted and the p-HSP20 and np-HSP20 forms were separated on one dimensional isoelectric focussing gels and detected by western blot using HSP20 antibody that recognize both forms. Panel A: Representative western blot of p-HSP20 and np-HSP20. Panel B: Cumulative data representing the relative amounts of p-HSP20 over total HSP20, obtained when the phosphorylated and non-phosphorylated bands were quantitated densitometrically. n = 4, p<0.05. *significant compared to histamine.

Forskolin treatment of smooth muscle increases the phosphorylation of HSP20 and induces relaxation [33]. To decipher the role of HSP20 phosphorylation in the regulation of actin polymerization during force suppression, HSP20 phosphorylation was examined by isoelectric focusing and western blot analysis. As expected, forskolin led to increases in the phosphorylation of HSP20 when compared to untreated or histamine treated tissues. (0.77±0.09 p-HSP20/total HSP20 vs 0.07±0.02 and 0.03±0.01 for untreated and histamine, respectively, p<0.05, n = 4). Treatment with histamine after forskolin did not reverse HSP20 phosphorylation (0.70±0.03, Figure 5).

**Figure 6:**
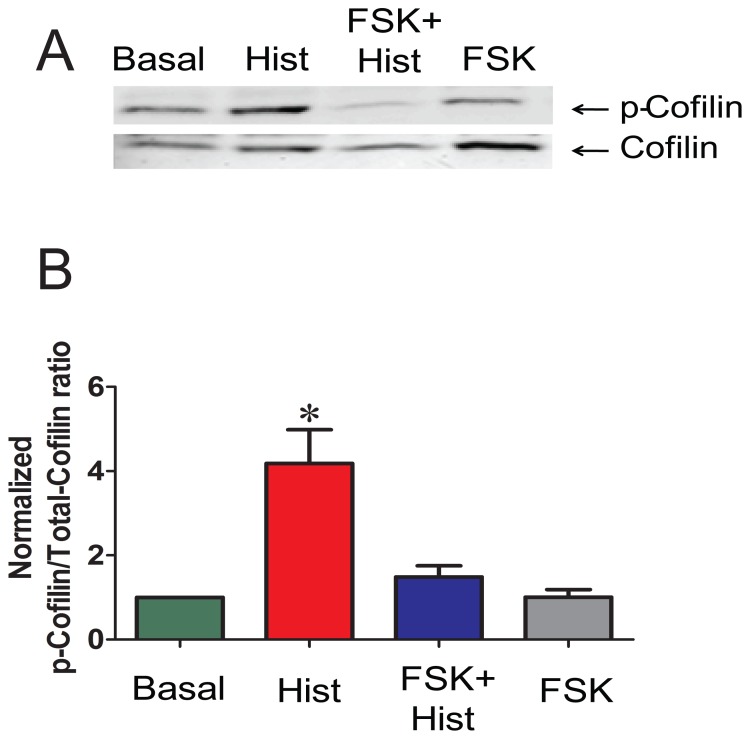
Preincubation with forskolin reduces phosphorylation of cofilin in PCA. PCA rings were treated with the same conditions as described in Figure 3. Proteins were extracted and separated on SDS polyacrylamide gels and p-cofilin was detected by western blotting. Panel A: Representative western blot of p-cofilin and np-cofilin. Panel B: Cumulative data representing the relative amounts of p-cofilin over the total cofilin and then normalized to the basal. Quantitative values were obtained when the phosphorylated and non-phosphorylated bands were quantitated densitometrically. n = 4, p<0.05. *significant compared to all other conditions.

**Figure 7:**
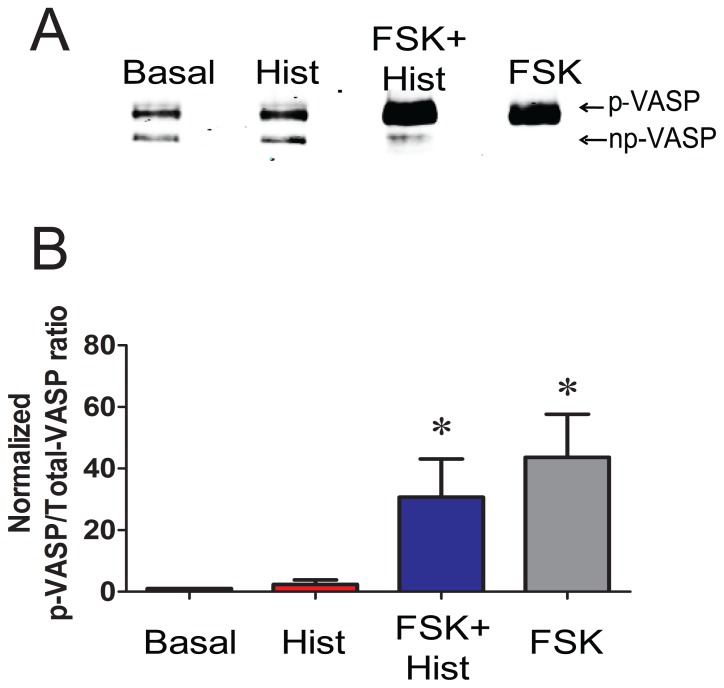
Preincubation with forskolin induces phosphorylation of VASP. Conditions are the same as described in Figure 3. Proteins were extracted and separated on SDS polyacrylamide gels. Phosphorylation of VASP causes mobility shift in the SDS gel and p- and np- VASP forms were detected by western blotting using VASP antibody that recognizes both forms. Panel A: Representative western blot of p- and np-VASP. Panel B: Cumulative data representing the relative amounts of p-VASP/total VASP and then normalized to the basal. Quantitative values were obtained when the phosphorylated and non-phosphorylated bands were quantitated densitometrically. n = 4, p<0.05. *significant compared to histamine.

Next, the effects of forskolin on cofilin and VASP phosphorylation, both of which have been demonstrated to regulate actin polymerization, were determined. Histamine increased the phosphorylation of cofilin in PCA by 2.9±1.2 fold, while forskolin pretreatment prevented histamine-induced increase in the phosphorylation of cofilin in PCA (4.2±0.8 and 1.5±0.3 p-cofilin/total cofilin for histamine and forskolin plus histamine, respectively (*p*<0.05, n = 4, Figure 6). Histamine treatment did not increase the phosphorylation of VASP (2.3±1.4 p-VASP/totalVASP). VASP was phosphorylated in response to forskolin (36.5±13.5 p-VASP/totalVASP, compared to the basal value p<0.05), and the phosphorylation was not significantly changed by histamine treatment after forskolin (25.8±11.3 p-VASP/total VASP, for forskolin plus and histamine, respectively, p<0.6 n = 6, Figure 7).

**Figure 8:**
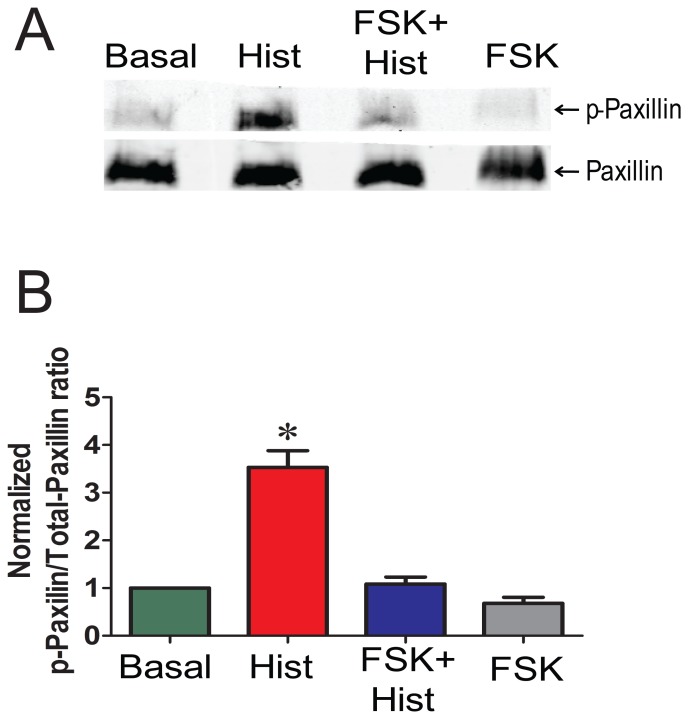
Preincubation with forskolin abolish histamine induced paxillin phosphorylation in PCA. Conditions are the same as described in Figure 3. Proteins were extracted and separated on SDS polyacrylamide gels and p-paxillin and np- paxillin were detected by western blotting. Panel A: Representative western blot of p-paxillin and np-paxillin. Panel B: Cumulative data representing the relative amounts of p-paxillin over total paxillin and normalized to the basal. Quantitative values were obtained when the phosphorylated and non-phosphorylated paxillin bands were quantitated densitometrically n = 4. p<0.05. *significant compared to all other conditions.

The cytoskeletal protein, paxillin, has been shown to regulate actin polymerization during contractile activation of smooth muscle [18], [34]. Hence, we studied the phosphorylation of paxillin during forskolin-induced suppression of force. Forskolin pretreatment prevented the phosphorylation of paxillin associated with histamine stimulation (3.5±0.7 and 1.1±0.1, p-paxillin/total paxillin, for histamine and forskolin plus histamine, respectively, p<0.05, n = 4, Figure 8), suggesting that inhibition of paxillin phosphorylation interferes with the actin polymerization needed for contraction.

## Discussion

In this study, a physiologic model system was developed wherein [Ca^2+^]_i_ was uncoupled from force, resulting in the complete suppression of force generation in the presence of a contractile agonist. Our approach is based on the premise that actin cytoskeletal regulation is an active component of cyclic nucleotide-dependent relaxation or inhibition of force. Utilizing PCA, we showed that activation of adenylyl cyclase by forskolin *prior* to histamine exposure completely suppressed half maximal force generated by histamine. This physiologic model allowed us to further define the role of actin filaments, in addition to thick filament modulation, during cyclic nucleotide-dependent force suppression. Experiments were conducted using the FluoroPlex Tissue Bath Fluorometry System, a unique muscle bath system that enables fluorescence ion recording in parallel with force measurement in intact tissues, and we measured [Ca^2+^]_i_ transients, MLC phosphorylation, F/G- actin levels, along with the phosphorylation of actin regulatory proteins.

While histamine-induced force generation was completely suppressed with forskolin pretreatment, a transient increase in [Ca^2+^]_i_ was still present (Figure1) suggesting that additional mechanisms to the reversal of activation involving decrease in [Ca^2+^]_i_ and crossbridge-dephosphorylation play a role in the suppression of force. However, we observed that the magnitude of the change in fluorescence ratio was significantly greater for tissue contracted with histamine when compared to tissue treated with forskolin followed by histamine (Figure 1 E). The time it took for the [Ca^2+^]_i_ to increase from 10% to 90% of its maximum response was significantly lower for tissue that was treated with histamine alone compared to forskolin followed by histamine treatment (data not shown), suggesting that the decoupling between [Ca^2+^]_i_ and force was not complete. It is possible that these changes may be due to the direct effect of forskolin on the [Ca^2+^]_i_. The effect was dose-dependent as the lower forskolin concentrations (1 µM) did not abolish the transient [Ca^2+^]_i_ or the force, and at a higher concentration (10 µM) there was no transient increase in [Ca^2+^]_i_ and force was completely suppressed. These results suggest that a higher forskolin dose inhibits the agonist-induced Ca^2+^ signaling pathways, possibly due to cross activation of PKG by cAMP, as reported by several investigators [35], [36], [37], [38]. Activation of PKG can reduce both the concentration of [Ca^2+^]_i_ and the force developed for a given intracellular [Ca^2+^] (i.e., the Ca^2+^ sensitivity) [39], [40]. Preliminary experiments also showed that pretreatment with the nitric oxide donor, sodium nitroprusside, not only completely blocked histamine-induced increase in [Ca^2+^]_i_ and force but also decreased the phosphorylation of MLC (Komalavilas *et al*., unpublished results).

Pretreatment of PCA with 5 µM forskolin completely suppressed histamine-induced contraction without significantly affecting histamine-induced changes in MLC phosphorylation (Figure, 2,3). This is similar to the results obtained in swine carotid artery where forskolin-induced force suppression in histamine pre-contracted tissue occurs without a reduction in MLC phosphorylation through a mechanism that involves regional actin filament inhibition or weak inhibition of myosin binding at the thin or thick filament. [41]. However, we observed that treatment of PCA with 10 µM forskolin prior to histamine treatment significantly reduced the MLC phosphorylation suggesting activation of PKG-regulating Ca^2+^ regulatory pathways. PKG activates MLC phosphatase, thereby reducing MLC phosphorylation [39].

Actin polymerization occurs in response to contractile stimuli in many smooth muscle tissues, and force development can be significantly reduced by treatment with inhibitors of actin polymerization (reviewed in [16], [17], [42]). Similar to prior reports using dog trachealis [43] and swine carotid artery [41], histamine increased F-actin concentration in PCA (Figure 4). Forskolin pretreatment prevented the histamine-induced increase in F-actin (Figure 4 A, B). This can be explained by the observation that forskolin treatment alone reduced basal F-actin concentration, suggesting cyclic-nucleotide-mediated actin depolymerization preceded any histamine-mediated cytoskeletal reorganization. We showed that reduction in F-actin was evident in the higher (5 and 10 µM) doses of forskolin tested. However, treatment of PCA with 1 µM forskolin did not completely inhibit the force or prevent the histamine induced increase in F-actin (Figure 2C). This result is in agreement to the Meeks *et al* study where no significant change in the F-actin levels was observed after forskolin (1 µM) treatment of histamine-contracted tissue [41]. It may be that forskolin induced reduced force despite increased MLC phosphorylation require only HSP20 phosphorylation while complete suppression of force with higher dose of forskolin involve HSP20 phosphorylation as well as decrease in F-actin level. It is also possible that the change in F-actin level is only detectable with the assay used when the force is completely suppressed by the use of a higher dose of forskolin, and that the subtle changes in F-actin level may not be detectable while there is only partial decrease in force using a lower dose of forskolin. Forskolin-induced suppression of force was reversible as washing the rings repeatedly allowed the PCA to contract to 5 µM histamine in a time-dependent manner (Figure 1F). Several investigators have demonstrated that latrunculin and cytochalasin, agents known to decrease actin polymerization, inhibit agonist-induced contraction [44], [45], [46]. However, these agents affect force by direct alteration of contractile filaments, while this study describes second messenger-mediated regulation of actin cytoskeletal-associated protein function.

A number of actin-associated proteins can be phosphorylated upon PKA or PKG activation. One such protein is HSP20, which can be phosphorylated on serine (Ser16) upon PKA or PKG activation leading to relaxation or force suppression independent of MLC phosphorylation [8], [22], [23]. HSP20 phosphorylation mediates relaxation or suppression of force through mechanisms that involve actin cytoskeletal regulation. Forskolin at 1 µM did not significantly change the MLC phosphorylation but increased the phosphorylation of HSP20 suggesting that phosphorylation of HSP20 alone partially reduced the force generated by histamine, possibly through mechanisms other than actin depolymerization. This also suggests that complete suppression of force requires higher doses of forskolin which not only increase the phosphorylation of HSP20 but also decrease F-actin. One mechanism involves interaction of p-HSP20 with scaffolding protein 14-3-3 and the actin depolymerizing factor cofilin, which causes depolymerization of actin resulting in deactivation of actin cytoskeleton and relaxation [24]. Forskolin treatment also decreased the phosphorylation of cofilin in the model described here (Figure. 6). Cofilin in its phosphorylated form binds to the intracellular scaffolding protein, 14-3-3 [47]. When displaced from 14-3-3, cofilin becomes dephosphorylated and acts as an actin depolymerizing protein [48], [49]. Phosphorylated HSP20 formed a tight complex with 14-3-3 in which dimer of 14-3-3- was bound to dimer of HSP20 [50]. Moreover, binding of 14-3-3 protein to p-HSP20 peptide prevented the association of cofilin with 14-3-3 [24]. This suggests that, forskolin treatment leads to phosphorylation of HSP20 which then binds to 14-3-3 and displaces cofilin. Consequently, the displaced cofilin is dephosphorylated leading to activation of cofilin as an actin depolymerization factor resulting in actin depolymerization [24], [25]. HSP20 also has a sequence homology with troponin 1 and a peptide containing this homology bound to actin filaments, reducing actin–activated myosin ATPase activity. This mechanism leads to the relaxation of skinned smooth muscle by partial or full inhibition of local myosin binding at the thin-or thick filament level [23], [41]. Phosphorylated HSP20 has been shown to promote airway smooth muscle relaxation, possibly through depolymerization of F-actin as well as inhibition of myosin binding to actin [25], [51]. In colonic smooth muscle, HSP20 is activated by PKA and modulates the association of caldesmon and tropomyosin during the maintenance of tone [52]. HSP20 may act as the functional switch for contraction and relaxation in the colonic smooth muscle [52]. HSP20-mediated force suppression may indeed involve different mechanisms in smooth muscle from various types and species.

In this study, forskolin also increased the phosphorylation of VASP, and the phosphorylation was not reversed upon histamine stimulation (Figure 7). Phosphorylation of VASP has been shown to be involved in the regulation of actin polymerization and decreases in the affinity of VASP for actin by 40 fold [53], [54]. VASP knockdown experiments have demonstrated that VASP-mediated elongation of actin filaments are necessary for vascular contractility and that VASP phosphorylation is decreased during phenylephrine induced force generation [42]. VASP phosphorylation by PKA has a negative effect on actin nucleation, and may act as a negative regulator of actin dynamics [55]. Our results are consistent with this model in that forskolin-induced phosphorylation of VASP, affecting histamine-induced actin polymerization, thus preventing force generation.

This study demonstrated that histamine-induced paxillin phosphorylation was prevented by forskolin pretreatment (Figure 8). Paxillin is a focal adhesion protein that is proposed to link actin filaments to integrin rich cell adhesion sites and may be involved as a cross-linker between the thin filaments and the dense bodies [29], [56]. Paxillin phosphorylation has been associated with the coordinated formation of focal adhesions and stress fibers [57]. Paxillin phosphorylation was increased during acetylcholine-induced contraction of intact trachealis smooth muscle and has been shown to play an essential role in regulating smooth muscle contraction [29]. Gunst *et al* has proposed that contractile agonists activate FAK, inducing tyrosine (118) phosphorylation of paxillin, causing CrkII coupling and formation of the CrkII/Cdc42/N-WASp complex, leading to the activation of Cdc42, N-WASp and Arp2/3 complex and actin polymerization [58]. In swine carotid artery stimulated with high-K^+^ or histamine, paxillin phosphorylation (Y118) and actin polymerization were increased only after full force development suggesting a role for paxillin phosphorylation during the sustained contraction [18], [29]. Stimulus-induced tyrosine phosphorylation of paxillin is associated with increases in actin polymerization in different smooth muscle tissues (reviewed in [59]). Although paxillin is not a substrate of PKA, its phosphorylation is affected by the activation of cAMP pathway indirectly, possibly by cross talk between the tyrosine and serine/threonine kinase pathways or that an intact actin cytoskeleton is necessary for paxillin phosphorylation. Hence, inhibition of histamine-induced paxillin phosphorylation by the activation of the cAMP pathway in PCA may indeed contribute, in part, to the decreased actin polymerization observed in our model, thereby inhibiting force.

### Conclusions

A physiological model was developed in which forskolin pretreatment completely suppressed histamine-induced force in PCA by regulating actin polymerization and dynamics without abolishing increases in [Ca^2+^]_i_ or MLC phosphorylation. Actin depolymerization creates conditions in which there is no actin filament structure on which MLC can treadmill. Thus, calcium desensitization is likely not the only mechanism of force modulation during activation of cyclic nucleotide-dependent signaling pathways. Our results further suggest that the actin cytoskeletal changes are mediated by the phosphorylation changes of actin modulatory proteins, such as HSP20, cofilin, VASP and paxillin. The force suppression model employed in this study can be used to further characterize the role of actin and its associated proteins in the regulation of vascular smooth muscle tone. Future studies may also examine later events (3–30 min) of force maintenance. In addition, cyclic nucleotide analogues can be used to decipher the different mechanisms that contribute to these regulatory pathways.
